# Supplementary material for: Mesenchymal Stem Cells and MSCs-Derived Extracellular Vesicles in Infectious Diseases: From Basic Research to Clinical Practice
Source: Bioengineering (Basel). 2022 Nov 8;9(11):662. doi: 10.3390/bioengineering9110662 (PMC9687734; doi:10.3390/bioengineering9110662)

Supplementary materials:

The following are available online at www.mdpi.com/xxx/s1, Figure S1: Growth of the global stem cell therapy market.

Figure S1. Growth of the global stem cell therapy market (https://www.researchandmarkets.com/content-images/233/233316/2/global-stem-cell-market.png).

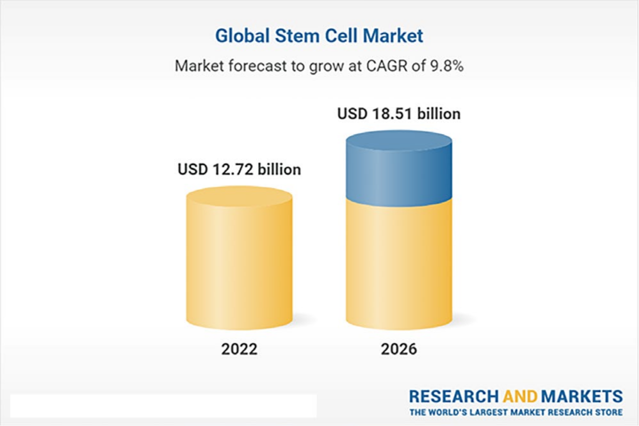

Supplement: Supplementary file 1 [file bioengineering-09-00662-s001.zip › bioengineering-1966156-supplementary.pdf]
